# Trauma and perceived social rejection among Yazidi women and girls who survived enslavement and genocide

**DOI:** 10.1186/s12916-018-1140-5

**Published:** 2018-09-13

**Authors:** Hawkar Ibrahim, Verena Ertl, Claudia Catani, Azad Ali Ismail, Frank Neuner

**Affiliations:** 1 Department of Psychology, Clinical Psychology and Psychotherapy, Bielefeld University, Bielefeld, Germany; 2 Department of Clinical Psychology, Koya University, Koya, Kurdistan Region Iraq; 3 vivo International, Konstanz, Germany

**Keywords:** Kurd, Yazidi, Genocide, Enslavement, PTSD, Depression, Perceived social rejection

## Abstract

**Background:**

In August 2014, the Islamic State of Iraq and Syria (ISIS), a terrorist organization, attacked the Yazidi’s ancestral homeland in northwestern Iraq. Among other atrocities, they abducted thousands of women and girls and traded many of them into sexual slavery. The aim of this study is to determine the mental health of women and girl survivors of these events in relation to enslavement and experiences with genocide-related events, as well as perceived social rejection in their community.

**Methods:**

Between February and July 2017, trained local assessors interviewed a sample of 416 Yazidi women and girls (65 of whom had survived sexual enslavement), aged between 17 and 75 years, and living in internally displaced person camps in the Kurdistan Region of Iraq. Post-traumatic stress disorder (PTSD) and depression symptoms were assessed using validated Kurdish versions of standard instruments. Scales for trauma exposure and perceived rejection were developed for the purpose of this study.

**Results:**

Participants reported a high number of traumatic events. More than 80% of girls and women, and almost all participants who were formerly enslaved, met criteria for a probable DSM-5 PTSD diagnosis. Trauma exposure and enslavement predicted poor mental health. In addition, among formerly enslaved girls and women, perceived social rejection in their community mediated the relationship between traumatic enslavement events and depression symptoms.

**Conclusions:**

In a context of maximum adversity, enslavement and war-related events contribute to high levels of PTSD and depression. Perceived social rejection seems to play a role in the relationship between trauma exposure and mental health among abducted genocide survivors. Providing psychosocial support and treatment for Yazidi people is essential and urgently required.

**Electronic supplementary material:**

The online version of this article (10.1186/s12916-018-1140-5) contains supplementary material, which is available to authorized users.

## Background

The Yazidis (*Êzidî*) are a Kurdish religious minority living in the north of Iraq, western Iran, eastern Turkey, and northern Syria [1]. They are followers of Yazidism, a non-Abrahamic, orally transmitted religion [2] that shares common characteristics with Christianity, Islam, and other monotheistic religions. Following the upheaval of the Arab Spring movement in the Middle East, an Islamic fundamentalist militant group, the so-called Islamic State of Iraq and Syria (ISIS), declared an Islamic Caliphate in Syria and Iraq. In June 2014, ISIS fighters captured the center of the Nineveh governorate in Iraq and announced a campaign to purify their Caliphate of non-Arab and non-Sunni Muslim communities, committing numerous atrocities against the civilian population. Due to their ethnicity and religion, Yazidis, as a Kurdish religious minority, were among the most severely affected communities [3]. In August 2014, ISIS attacked the Yazidi’s ancestral homeland in northwestern Iraq, close to the Iraqi–Syrian border. During the attack, ISIS killed, kidnapped, and enslaved thousands of children, men, women, and girls, displacing the entire community to refugee camps in the process [4]. Based on survey data, Cetorelli et al. [5] estimated that 3100 Yazidis were killed and 6800 were kidnapped in this operation. The Independent International Commission of Inquiry on the Syrian Arab Republic by United Nations Human Rights Council investigated the violations committed against Yazidis and documented that the Yazidi people were subjected to mass killings, rape, sexual violence, enslavement, torture, and forcible transfer, leading the it to declare ISIS’s crimes against the Yazidis as a genocide [6].

War and atrocities in the context of genocide have negative effects for the survivors at both the individual and collective levels. Multiple types of traumatic events during periods of genocide, including witnessing extreme violence, the disappearance and loss of family members, rape and sexual humiliation, torture, imprisonment, and kidnapping [7, 8], can have psychological consequences [9–11]. Research conducted in other conflict regions, including Rwanda [12] and Bosnia [13], has found that genocidal atrocities bring about long-lasting and severe effects for the survivors, with up to almost 70% of the survivors fulfilling criteria for trauma-related disorders. Studies among non-Yazidi Kurdish genocide survivors in the Middle East have shown that survivors still suffer from a wide range of mental health conditions years and even decades after the genocide campaigns [14–17]. A few recent studies with small- to medium-sized samples of forcibly displaced Yazidis have documented high rates of mental ill-health. Based on clinical interviews, Tekin et al. [18] found rates of 43% for PTSD and 40% for major depression among Yazidis displaced into Turkey. Similar levels were found for Yazidi children in Turkey [19, 20].

Sexual violence against women has commonly been systematically used during wars and genocide, with the aim of traumatizing the civilian population and the elimination of the targeted group through the desecration of individual group members [21]. Systematic rape and sexual violence have devastating effects on social, psychological, and physical health, including genital and non-genital injuries experienced by the survivors [22, 23]. Such violence contributes to a range of psychological disorders. Clinically significant psychological disorders have been documented in 69.4% of survivors of war-related sexual violence in northern Uganda. Sexual violence may occur over extended periods of time after the abduction of girls and women. Such extreme adversity hardly goes without long-term harm for the survivors, with almost 85% of the sample of abducted rape survivors from Bosnia and Congo presenting with trauma-related disorders [24]. A recent review of 20 studies of civilians who experienced war-related sexual violence from six countries across Africa and Europe concluded that the psychological sequelae of wartime sexual violence most often included extreme rates of PTSD, anxiety, and depression [25]. More recently, Hoffman et al. [26] assessed the prevalence of PTSD as well as complex PTSD among 108 female Yazidi former ISIS captives and found that 50.9% of them had probable complex PTSD, while 20% had probable PTSD.

The negative impacts of rape and wartime sexual violence extend into the survivors’ social lives. Victims of wartime sexual violence are commonly faced with rejection by their community and family members [27, 28]. Sexual violence has been associated with perceived levels of stigma and poor community relations among girls who were abducted by a rebel army in northern Uganda [29]. A recent study in the war region of eastern Congo [30] documented stigmatization, rejection, and abandonment among survivors of sexual violence. More than half of survivors of sexual violence had been told they should leave their home because they had been raped, and the same proportion perceived that their status in the community had decreased. More than two-thirds of survivors avoided attending church due to fear of being stigmatized as a survivor of sexual violence.

It is likely that the psychological and social consequences of sexual violence are more than independent outcomes that occur on different socioecological levels. Across different conceptual and theoretical frameworks, the association between social factors and psychological trauma has been well documented. Social support from the immediate environment has been identified as one of the most consistent predictor of psychological adaptation following a wide range of traumatic event types [31], including forced displacement [32, 33], although causality of this relationship remains unclear [34, 35]. Consistent with research on social support, the manner and extent to which people in the social community acknowledge the survivor’s experiences of violence are associated with the survivor’s well-being [36–39]. Conversely, social rejection seems to promote and maintain the symptoms of psychological disorders. A significant association between family rejection, PTSD, and depression symptoms has been documented among conflict-affected adult women in the eastern Congo [40]. A similar association between social discrimination and a range of mental health problems was also found in formerly abducted girls in northern Uganda [41].

While the negative psychosocial consequences of war-related mass sexual violence seem obvious given the background of current knowledge about trauma, there is a dearth of systematic research on individuals who experienced extreme levels of adversities during war, including enslavement. Herein, we compared two samples of Yazidi survivors of the ISIS atrocities in the Middle East, one with a history of enslavement and one without experiences of such. In this context, we aimed to determine predictors for poor mental health, specifically seeking to investigate whether enslavement has a unique contribution to PTSD and depression symptoms above and beyond other traumatic, war-related events. In addition, given the observation that some of the formerly abducted survivors reported rejection from their own communities, we aimed to test whether perceived social rejection contributed to the maintenance of poor mental health.

## Methods

### Participants

The participants consisted of 416 female Yazidi adult and youth survivors of the civil war in Syria and northern Iraq. A portion of them (15.6%) were survivors who had been abducted by ISIS and kept as slaves. Their periods of abduction and enslavement ranged from 1 day to 2.5 years (*M* = 9.01, *SD* = 9.21, in months). At the time of the interview, the age of participants ranged between 17 and 75 years (*M* = 31.68, *SD* = 12.63). The majority (78.4%) were currently married and more than half (54.3%) were illiterate. Only 10.4% of the married participants had no children, while the other married participants had between 1 and 12 children (*M* = 3.26, *SD* = 3.12). While a large majority of the participants (87.7%) had no regular monthly income, others had monthly incomes between 70,000 and 900,000 IQD (1390 IQD = 1 €, local rate). Only 18.5% of participants reported having received any type of psychosocial support (Table 1).

**Table 1:** Sociodemographic information and traumatic experiences

	Total sample (*n* = 416)	Non-slaves (*n* = 351)	Formerly enslaved (*n* = 65)
Age, mean (SD)^a^	31.68 (12.63)	30.48 (11.15)	38.16 (17.42)
Current marital status, *n* (%)			
Single	90 (21.6)	79 (22.5)	11 (16.9)
Married	326 (78.4)	272 (77.5)	54 (83.1)
Formal education, mean (SD)^a^	2.78 (3.69)	2.9 (3.74)	2.12 (3.40)
Place of growing up, *n* (%)			
Town	160 (38.5)	135 (38.5)	25 (38.5)
Village	256 (61.5)	216 (61.5)	40 (61.5)
Occupation, *n* (%)			
Currently working	26 (6.3)	18 (5.1)	8 (12.3)
Currently not working	390 (93.8)	333 (94.9)	57 (87.7)
Having regular income, *n* (%)			
No	365 (87.7)	311 (88.6)	54 (83.1)
Yes	51 (12.3)	40 (11.4)	11 (16.9)
Individual monthly income, mean (SD)^b^	27,824.51 (105,596.48)	25,470.08 (100,864.08)	40,538.46 (128,349.26)
Number of children, mean (SD)	3.26 (3.12)	3.19 (3.17)	3.60 (2.86)
Number of boys, mean (SD)	1.65 (1.83)	1.62 (1.84)	1.81 (1.75)
Number of girls, mean (SD)	1.59 (1.76)	1.56 (1.76)	1.78 (1.76)
Number of lifetime displacements, mean (SD)	1.10 (0.5)	1.11 (0.53)	1.06 (0.24)
Age during war, mean (SD)^a^	28.96 (12.68)	27.68 (11.17)	35.87 (17.40)
Location during war			
Town	161 (38.7)	137 (39)	24 (36.9)
Village	255 (61.3)	214 (61)	41 (63.1)
Number of family members directly affected by ISIS, mean (SD)^c^	6.16 (12.71)	4.20 (10.43)	16.73 (17.83)
Receiving psychosocial support			
No	339 (81.5)	294 (83.8)	45 (69.2)
Yes	77 (18.5)	57 (16.2)	20 (30.8)
Traumatic events, mean (SD)^d^	5.79 (3.02)	5.79 (3.02)	12.15 (4.30)
War-related events mean (SD)^e^	3.40 (2.05)	3.40 (2.05)	7.38 (2.19)
General life events^f^	2.76 (2.37)	2.39 (2.01)	4.76 (3.08)

^a^ In years

^b^ In Iraqi Dinar

^c^ Score range: 0–140

^d^ Score range: 0–25

^e^ Score range: 0–10

^f^ Score range: 0–15

### Procedures

#### Sampling

The sample was drawn from Yazidi women and girls residing in the Khanke and Arbat camps for internally displaced people (IDP), located in the Dohuk and Sulaymaniyah Governorates of the Kurdistan Region of Iraq (KRI). Of the 436 Yazidi women and girls invited to participate in this study, 22 refused. The most commonly reported reasons for refusing were “*I don’t want to talk about this experience*” and “*I got tired of interviews*”. The participants were selected based on a pragmatic sampling approach. The camps were subdivided into six to seven sections by the camp administrations, and tents were chosen based on a random selection of households by spinning a pen from the zone center. Trained interviewers visited the household that was in a straight line from the tip of the pen, identified the girls and women in the household and determined their eligibility for participation in the study. From each nuclear family, a maximum of two women or girls of at least 17 years of age were interviewed. All participants were interviewed individually by using a structured interview based on standardized questionnaires.

#### Protection and safety

All participants were informed about the availability of no-cost mental health services inside their IDP camps. The address cards and contact details of relevant organizations were provided to educated participants. Before the start of data collection, we created a referral system in collaboration with local NGOs and camp administration for those participants who wished to receive psychological help and for those who were severely affected by displacement and enslavement events or by family issues. In Khanke camp, we referred our participants to the Rawshan center of People’s Development Organization, supported by Norwegian People’s Aid. Rawshan (*Rewşen*) is a People’s Development Organization community center in Khanke camp that provides services to the majority of displaced people. Rawshan has a specific focus on women and young girls, especially to those who suffer from mental health problems, family, and gender-based violence. Rawshan center’s mental health programs include psychiatric help provided by female psychiatrists, with psychotherapy and counseling provided by female psychologists trained at the BSc level.

In Arbat camp, we referred participants who needed support to the camp hospital to receive first aid psychiatric help or to NGOs that provided mental health rehabilitation programs. The location of the participants’ tent and NGO’s waiting list were taken into consideration when referring the individual to a specific NGO. In total, 32 participants (29 from Kahnke and 3 from Arbat camp) were referred for psychological/psychiatric help. Upon request of the participants, a 2-week follow-up was conducted by the first author, with the purpose of ensuring that the referred participants had received adequate support.

#### Interviewers

The interviews were conducted by 10 trained, local female BSc-level clinical psychologists who had experience with the displaced people living in KRI. As part of the training, intensive theoretical and practical training about the study instruments, mental health risk management, and diagnostic and ethical issues in mental health research studies were provided during a 7-day workshop. In addition, local interviewers also attended a 1-day tour to Arbat and Khanke camps to provide relevant information about the profile of the camps and to meet the administration staff and those local and international organizations who have psychosocial support programs based in the camps. The interviews took place in the participants’ tents and lasted between 60 and 90 min.

The population in the camp, particularly the illiterate participants, expressed a strong distrust and skepticism of any official authority figures and were not willing to sign any document. Due to the skepticism of the population and to protect the participants, we relied on obtaining verbal rather than written informed consent through reading standardized written consent information sheets to the respondents. Each explicit verbal consent was documented by the interviewer and confirmed by her signature. The study and its procedure, including the reliance on verbal informed consents, were approved by the Ethical Committee of Bielefeld University in Germany as well as the Ethical Committee of Koya University in the KRI.

### Instruments

#### Traumatic events

To examine general and war-related traumatic events experienced during the war, we developed the War and Adversity Exposure Checklist as a combination of items that had been collected from various sources, including existing trauma instruments, such as the War Exposure Scale [42], Life Events Checklist for DSM-5 [43], and informal interviews with war survivors from the Syrian and Iraqi civil war. Both the War Exposure Scale and the Life Events Checklist for DSM-5 had been previously employed in the validation study among Kurdish and Arab displaced populations living in the KRI [42]. The trauma score was computed by summing the affirmative answers to the items on the instrument. The internal consistency of the War and Adversity Exposure Checklist, as measured by Cronbach’s alpha, was acceptable (α = 0.77).

#### Enslavement trauma scale

This checklist was specifically developed for this study to assess potentially traumatic events that occurred during enslavement. It was created on the basis of open discussions with ISIS slave survivors and key informants, including psychological staff in the camps. The 20-item events reflected Yazidi women’s experiences of abuse by ISIS (e.g., forced religious conversion, being sold in ISIS sex slave markets, and witnessing people being beheaded or burnt to death, etc.). The checklist had a high internal consistency (Cronbach’s alpha = 0.90).

#### Perceived social rejection

In order to examine the social experience of survivors within their family and social community post-enslavement, a short questionnaire of perceived social rejection was developed based on interviews with survivors who had previously been abducted by ISIS. The wording of existing stigma and social rejection scales were too complex to employ them with the population included in this study due to the high rates of illiteracy. The resulting questionnaire consisted of five questions (‘Are you worried about not getting married or remaining married as result of what you have experienced?’, ‘Do you feel excluded by your family?’, ‘Do you feel excluded (or stigmatized) by members of your community?’, ‘Are you avoiding people or social situations (events) as a result of fear of being rejected or stigmatized?’, and ‘Are you worried about what other people think of what you have experienced?’) with responses rated on a 4-point Likert scale. Response scores for each item ranged from 0 to 3 (Not at all = 0, A little = 1, Quite a bit = 2, Extremely = 3). The items of the perceived social rejection scale were factor analyzed using maximum likelihood factoring with oblique rotation. The Kaiser–Meyer–Olkin measure of sampling adequacy was 0.738, thus exceeding the recommended value of 0.6. Bartlett’s test of sphericity was significant (*p* < 0.0001). The analysis produced one factor that explained 44.23% of the variance, and item loadings on this factor ranged between 0.753 and 0.559. This questionnaire had acceptable internal consistency (Cronbach’s alpha = 0.79).

#### PTSD symptoms

We assessed PTSD symptoms with the Kurdish version of the PTSD Checklist for DSM-5 (PCL-5) [42]. The PCL-5 [44] is a self-report measure developed on the basis of the DSM-5 symptom criteria for PTSD, which contains 20 items, categorized into four symptom clusters and rated on a 5-point Likert scale, with scores ranging from ‘Not at all’ = 0 to ‘Extremely’ = 4. For the probable diagnosis of PTSD, the authors of the original version suggested a cut-off score of 33 [44], while a calibration study with the Kurdish version indicated a cut-off of 23 for the optimum fit with a diagnosis [42]. The internal consistency of the PCL-5 in the current study (Cronbach’s alpha = 0.86) was quite close to the consistency in the validation study.

#### Depression symptoms

The second part of Hopkins Symptom Checklist-25 [45] was used to examine depression symptoms. This checklist had previously been used within Kurdish and Arab populations [42]. It consists of 15 items on a 4-point scale (‘Not at all’, ‘A little’, ‘Quite a bit’, and ‘Extremely’ rated 1 to 4, respectively). The mean scores range between 1 and 4, where higher scores indicate higher levels of symptoms. The internal consistency of Hopkins Symptom Checklist-25 in the current study was high (Cronbach’s alpha = 0.89).

### Data analysis

The collected data was analyzed using the Statistical Package for Social Sciences (SPSS-Mac version 25 and JMP 13). An exploratory data analysis was conducted to determine whether PTSD and depression scores were normally distributed, in addition to visual inspection of the histograms and a Q-Q plots. Results showed that the PTSD and depression scores were approximately normally distributed for non-enslaved, formerly enslaved, and across groups. The same exploratory data analysis was conducted for other variables and results showed that perceived social rejection and enslavement events were also normally distributed (Additional file 1: Table S1). T-tests were applied to determine group differences. Relationships between continuous variables were tested with bivariate Pearson correlations for normally distributed variables (PTSD, depression, enslavement events, and perceived social rejection), and with Spearman-rank correlations for continuous variables with non-normal distributions (age, education, number of children, income, number of lifetime displacement, and trauma score); the association between dichotomous variables and continuous variables were tested using point-biserial correlations. A mediation analysis was carried out based on a bootstrapping procedure using PROCESS macro version 3.0 [46] for SPSS to clarify the mediating role of perceived social rejection in the association between enslavement events and mental health. The bootstrap mediation analysis is a non-parametric method that can be applied to small and moderate sample sizes [47], regardless of the distribution of the sample. Direct and indirect effects of enslavement events on symptoms of depression and PTSD were estimated using a set of ordinary least squares regressions. Standardized estimates of the resulting path coefficients, as well as tests of significance for each path were calculated using two regressions (one for the mediator as the outcome and one for the chosen measure of symptoms as the outcome). We used bootstrapping based on 5000 bootstrap samples to infer statistical significance (alpha level = 0.05). To determine the unique effect size and statistical significance of potential predictors of PTSD and depression symptoms we calculated linear models. Although the intracluster correlations were generally low (PTSD symptoms intracluster correlation coefficient = 0.0053; depression symptoms intracluster correlation coefficient = 0.0054), we accounted for cluster sampling using a mixed linear model calculated with JMP 13 (SAS Institute, Cary, NC, USA) that provides mixed models based on the SAS PROC MIXED procedure. Internal consistency was determined using Cronbach’s alpha.

## Results

### Traumatic events

Participants reported experiencing between 0 and 20 traumatic events of different types (*M* = 5.79, *SD* = 3.02). Of all participants, 99% had experienced at least one traumatic event; 85.1% of participants reported that they had experienced food and water deprivation, 63.7% had direct exposure to armed- and combat-related events, and half of the participants were separated from their family members by force. Regarding general life events during the period of genocide, witnessing fire or explosion (43.5%), natural disaster (29.3%), and transportation accidents (26%) were among the most common traumatic life events. Formerly enslaved participants reported experiencing and/or witnessing a significantly higher number of traumatic events than did non-enslaved women and girls (non-enslaved: *M* = 5.79, *SD* = 3.02; formerly enslaved: *M* = 12.15, *SD* = 4.30; two-tailed *t* test (unequal variances): *t* (76.07) = − 11.38, *p* < .001). Furthermore, formerly enslaved participants had also experienced a significantly higher number of general and war-related traumatic events (war related events: (non-enslaved: *M* = 3.40, *SD* = 2.05; formerly enslaved: *M* = 7.38, *SD* = 2.19; two-tailed *t* test (equal variances): *t* (414) = − 14.215, *p* < 0.001); general life events: (non-enslaved: *M* = 2.39, *SD* = 2.01; formerly enslaved: *M* = 4.76, *SD* = 3.08; two-tailed *t* test (unequal variances): *t* (74.4) = − 7.94, *p* < 0.001).

### Enslavement events

**Fig. 1:**
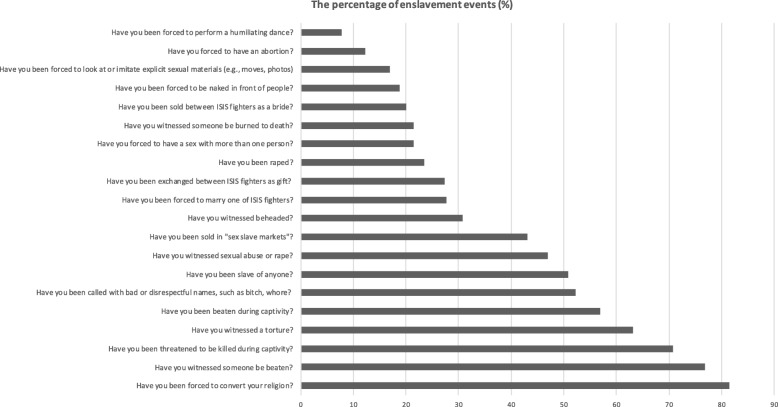
The percentage of enslavement events

As shown in Fig. 1, formerly enslaved participants experienced between 0 and 20 enslavement event types (*M* = 7.96, *SD* = 5.21). There was no statistically significant difference between women and girls in experiencing enslavement events (girls: *M* = 8.09, *SD* = 6.93; women: *M* = 7.61, *SD* = 4.86; two-tailed *t* test (equal variances): *t* (63) = − 0.276, *p* = 0.783). There was no statistically significant relationship between enslavement events and age (*p* > 0.05).

### Mental health symptomatology

Formerly enslaved subjects reported significantly higher levels of PTSD (*M* = 61.5 vs. 47.5) and depression (*M* = 45.3 vs. 35.7) symptoms than did non-enslaved women and girls (*p* < 0.001). Using cut-off scores of 33 as a preliminary cut-point score suggested for the instrument [44], 88.9% of non-enslaved and 98.5% of formerly enslaved participants met DSM-5 symptom criteria for PTSD. When we used the culturally validated cut-off score of 23 [42], we found that all formerly enslaved and 97.2% of non-enslaved participants fulfilled the DSM-5 criteria for a PTSD diagnosis.

### Perceived social rejection

Formally enslaved participants reported perceived social rejection (*M* = 7.04, *SD* = 4.93, range 0–15), with 32.3% being worried about not getting married or continuing in their marriage. The participants’ feeling of exclusion by family and community members was high; 44.6% of formerly enslaved participants felt extremely excluded by community members. In addition, 49.2% reported extreme worry about what people thought of what they had experienced and 40% of them reported that they avoided people or social events as a result of fear of being rejected or stigmatized. The levels of perceived social rejection were not correlated with participants’ level of education, age, or enslavement duration, but there was a significant positive relationship between perceived social rejection and the number of experienced enslavement events (Additional file 1: Table S2).

### Associations of enslavement events, mental health, and perceived social rejection

**Fig. 2:**
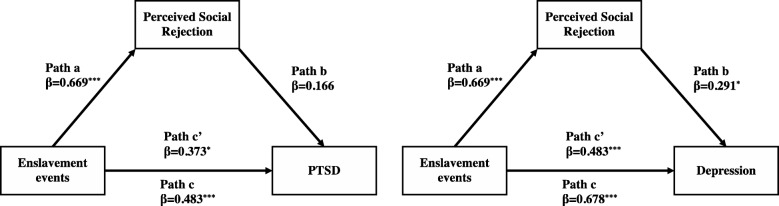
Mediating role of perceived social rejection in the association between enslavement events with PTSD and depression, controlling for number of experienced traumatic events. *p < 0.05, ***p < 0.001

As Fig. 2 illustrates, after controlling for the number of experienced traumatic events, the sum score of enslavement events was positively correlated with perceived social rejection (β = 0.669, *t* = 4.49, *p* < 0.001), PTSD (β = 0.483, *t* = 3.59, *p* < 0.001), and depression (β = 0.678, *t* = 5.88, *p* < 0.001). To examine the mediating role of perceived social rejection in this association we tested a simple mediation effect. After cntrolling for the mediating influence of perceived social rejection, the direct effect of enslavement events on PTSD and depression was reduced but remained significant. The indirect effect was tested using a bootstrap estimation approach and results showed that the indirect effect reached significance for depression (β = 0.194, 95% CI 0.01 to 0.37) but not for PTSD (β = 0.117, 95% CI − 0.10 to 0.36).

### Prediction of mental health symptomatology

Hierarchical linear model analyses were used to investigate potential predictors for PTSD and depression symptoms. The first model was created for the whole sample (*n* = 416). In this model, we entered age, years of formal education, marital status, individual monthly income, the number of family members who were directly affected by ISIS, trauma score, and enslavement as independent variables to predict PTSD and depression as dependent variables (Table 2). In addition, bivariate relationships were analyzed using zero-order correlations. The second model has been proposed to explain predictors of PTSD and depression among formerly enslaved participants (*n =* 65) (Table 3). The normality assumption of residuals for both models was checked using box plots and Q-Q plots, as well as Kolmogorov–Smirnov and Shapiro–Wilk tests. The results showed that standardized and unstandardized residuals of PTSD and depression scores in both models were normally distributed (Kolmogorov–Smirnov and Shapiro–Wilk *p* > 0.05).

**Table 2:** Predictors of PTSD and depression symptoms among the total sample (*n* = 416)

Predictor	PTSD^a^		Depression^b^	
	Standardized ß-coefficient	Zero-order correlation	Standardized ß-coefficient	Zero-order correlation
Age	− 0.010	0.070	0.056	0.198**
Education	− 0.035	− 0.013	− 0.021	− 0.049
Marital status	− 0.013	0.020	0.082	0.139**
Income	0.089*	0.007	0.071	0.008
Number of family members directly affected by IS	0.195***	0.278***	0.213***	0.319***
Trauma score	0.182**	0.295***	0.196***	0.308***
Enslavement	0.171**	0.351***	0.127*	0.339***

^a^ PTSD = (F (7,408) = 24.04, *p* < 0.000), with an *R*^*2*^ of 0.19

^b^ Depression = (F (7,408) = 15.62, *p* < 0.001), with an *R*^*2*^ of 0.21

*p = 0.05, **p < 0.01, ***p < 0.001

**Table 3:** Predictors of PTSD and depression among formerly enslaved women and girls (*n* = 65)

Predictor	PTSD^a^		Depression^b^	
	Standardized ß-Coefficient	Zero-order correlation	Standardized ß-Coefficient	Zero-order correlation
Age	− 0.119	− 0.189	0.059	− 0.042
Education	− 0.127	− 0.092	0.087	− 0.008
Marital status	0.026	− 0.005	0.183	0.152
Income	0.026	0.059	− 0.057	− 0.013
Number of family members who directly affected by ISIS	0.244*	0.415**	0.173	0.479***
Trauma score	0.019	0.212	− 0.040	0.315**
Enslavement events score	0.308*	0.509***	0.642***	0.669***

^a^ PTSD = (F (7,57) = 3.14, *p* < 0.05), with an *R*^*2*^ of 0.27

^b^ Depression = (F (7,57) = 7.96, *p* < 0.001), with an *R*^*2*^ of 0.49

*p < 0.05, **p < 0.01, ***p < 0.001

## Discussion

The current study demonstrated the psychosocial consequences of genocide and enslavement among Yazidi women and girls living in IDP camps in KRI. Findings suggest that high rates of mental health symptoms were mainly predicted by the intensity of trauma exposure. Enslavement predicted a worse outcome over and above the effect of traumatic event types. At the same time, our findings indicate that perceived social rejection plays a mediating role in the relationship between trauma and mental health.

Yazidi women who survived war atrocities represent a highly traumatized population. Rates of trauma exposure documented in our study are in line with numerous reports by international organizations about sexual and gender-based crimes against Yazidi women and girls [3, 6, 48]. It is also consistent with studies among female survivors of other genocides and armed conflicts [49–51]. The high exposure to adversities in this population is associated with very high rates of mental ill-health, which confirms previous reports of excessive rates of mental disorders in extremely traumatized war populations [52–54]. Yazidi women and girls who survived enslavement reported even more severe PTSD and depression symptoms. This effect remained stable after controlling for traumatic event types experienced by the survivors. While we are not aware of research on comparable populations reporting enslavement, studies with survivors of abduction, including victims of sex trafficking [55], child soldiers [56, 57], and formerly abducted people [58, 59], have confirmed the extraordinarily harmful effects of abduction. Even when considering the severity of trauma load reported by the population, the prevalence rate of DSM-5 PTSD of approximately 90% found in this study is exceptionally high, especially when considering that a validated instrument was utilized. Such high prevalence of mental health disorders can be potentially attributed to the fact that all participants were females. Studies among genocide-affected populations showed that the prevalence rates of PTSD and depression are more than two times higher in women than in men [60, 61]. Moreover, subjects still lived in dependence and insecurity in refugee camps and less than one-quarter of the participants reported any type of professional psychosocial support. Furthermore, given that the Yazidis have a male-dominated and community-oriented culture, any intimate relationship outside of their social community is prohibited. Therefore, Yazidi women and girls who have a history of enslavement, rape, and sexual violence may find themselves isolated in the aftermath of enslavement, and this may contribute to severe mental health symptoms. Together, all these factors could be a potential explanation for the high prevalence rates of mental health disorders in this study’s sample.

Formerly enslaved women and girls perceived diverse levels of social rejection by their family and community members. The same phenomenon has been found among formerly abducted girls [62] and female victims of war-related sexual violence [27], who were likely to face or perceive stigma, discrimination, and social rejection. In line with research from Africa [41, 63], we found a significant relationship between mental health disorders and post-enslavement social stressors such as perceived stigma and social rejection. This finding is also consistent with results from meta-analytic studies that showed that, in general, perceived discrimination has negative outcomes on individual well-being [64, 65]. Furthermore, our findings indicate that the relationship between enslavement events and depression are partially mediated by perceived social rejection, while the mediation effect for PTSD did not reach significance. The nature of depression, associated with social conditions and life events, in contrast to PTSD, which may be conceptualized as a disorder of memory, could explain part of this finding.

This study has implications for the development of psychosocial and mental health programs. The high rates of mental health symptoms present should serve as a call to local and international organizations for urgent psychological intervention for Yazidi women and girls. Moreover, organizations could consider designing some social activity programs using the context of education for reintegrating formally enslaved females into their social community.

While our study is, to our knowledge, one of the first comprehensive studies to evaluate the mental health of Yazidi women and girls in the aftermath of genocide, several limitations should be noted. First, although we were careful to obtain an unbiased sample, it is impossible to evaluate to what extent the sample is representative of all female Yazidi survivors. Our sample consists only of those Yazidi women and girls who live in IDP camps in KRI, while some of the Yazidi survivors, especially those who were without male protection, live outside the camps. Further, although the majority of formally enslaved Yazidis were children and adolescents, we only interviewed those who were above the age of 17. Third, the results were also limited by the cross-sectional design, which prevents us from drawing temporal or even causal relationships in the interplay between traumatic events, social factors, and mental health outcomes. For example, it could be that PTSD and/or depressive symptoms mediate the effect on trauma exposure and perceived social rejection. Further longitudinal research could provide clarity to the links between these variables. Fourth, social rejection has been evaluated according to participants’ perception and, given that the majority of them are suffering from severe PTSD and depression symptoms, this may have an impact on the manner in which they perceive social reactions. Fifth, while participants had experienced repeated and multiple traumatic events, the current study only addressed PTSD, which is usually caused by a single traumatic event limited in duration. Complex PTSD, on the other hand, is a psychological syndrome following prolonged and multiple trauma.

## Conclusion

Our results scientifically documented that Yazidi women and girls had experienced genocide and other instances of suppression and oppression by ISIS, with little action from the rest of the world to support them. The present study illustrates the devastating psychological consequences of genocide and enslavement. Our findings call for urgent psychosocial intervention for Yazidi survivors of genocide.

## Additional file

Additional file 1: **Table S1.** Skewness and kurtosis for dependent and independent variables. **Table S2.** Intercorrelation between PTSD, depression, and traumatic events with demographic variables. (DOCX 25 kb)

## Abbreviations

IDP: internally displaced person
ISIS: Islamic State of Iraq and Syria
KRI: Kurdistan Region of Iraq
PCL-5: PTSD Checklist for DSM-5
PTSD: post-traumatic stress disorder
