# Supplementary material for: Trauma and perceived social rejection among Yazidi women and girls who survived enslavement and genocide
Source: BMC Med. 2018 Sep 13;16:154. doi: 10.1186/s12916-018-1140-5 (PMC6136186; doi:10.1186/s12916-018-1140-5)
Supplement: Supplementary file 1 — Table S1. Skewness and kurtosis for dependent and independent variables. Table S2. Intercorrelation between PTSD, depression, and traumatic events with demographic variables. (DOCX 25 kb) [file 12916_2018_1140_MOESM1_ESM.docx]

Additional file 1

| Table 1 Skewness, and kurtosis for dependent and independent variables | | | | | |
| --- | --- | --- | --- | --- | --- |
| Variables | | Skewness | SE Skewness | Kurtosis | SE Kurtosis |
| PTSD | Non-enslaved | .076 | .130 | -.139 | .260 |
|  | Formerly enslaved | -.198 | .297 | -.883 | .586 |
|  | Across groups | .06 | .12 | -.30 | .23 |
| Depression | Non-enslaved | .168 | .130 | -.555 | .260 |
|  | Formerly enslaved | -.354 | .297 | -.883 | .586 |
|  | Across groups | .17 | .12 | -.64 | .23 |
| Trauma score | Non-enslaved | .663 | .130 | .143 | .260 |
|  | Formerly enslaved | -.271 | .297 | -.465 | .586 |
|  | Across groups | .928 | .120 | .543 | .239 |
| Age | Non-slaves | 1.268 | .130 | 1.623 | .260 |
|  | Formerly enslaved | .819 | .297 | -.754 | .586 |
|  | Across groups | 1.345 | .120 | 1.495 | .239 |
| Education | Non-slaves | 1.051 | .130 | .163 | .260 |
|  | Formerly enslaved | 1.596 | .297 | 1.540 | .586 |
|  | Across groups | 1.119 | .120 | .287 | .239 |
| Number of Children | Non-slaves | .724 | .130 | -.553 | .260 |
|  | Formerly enslaved | .372 | .297 | -.624 | .586 |
|  | Across groups | .670 | .120 | -.584 | .239 |
| Income | Non-slaves | 4.686 | .130 | 23.557 | .260 |
|  | Formerly enslaved | 5.178 | .297 | 32.115 | .586 |
|  | Across groups | 4.869 | .120 | 26.946 | .239 |
| Number of life time displacement | Non-slaves | 7.190 | .130 | 64.255 | .260 |
|  | Formerly enslaved | 3.736 | .297 | 12.335 | .586 |
|  | Across groups | 7.487 | .120 | 71.276 | .239 |
| Enslavement Events | | .726 | .297 | -.540 | .586 |
| Perceived Social Rejection | | .121 | .297 | -1.208 | .586 |

| Table S2 Intercorrelation between PTSD, depression and traumatic events with demographic variables. | | | | | | | | | | | | |
| --- | --- | --- | --- | --- | --- | --- | --- | --- | --- | --- | --- | --- |
| Variables | 1 | 2 | 3 | 4 | 5 | 6 | 7 | 8 | 9 | 10 | 11 | 12 |
| 1.PTSD score | ____ |  |  |  |  |  |  |  |  |  |  |  |
| 2.Depression score | .723** | ____ |  |  |  |  |  |  |  |  |  |  |
| 3.Age | .070 | .198** | ____ |  |  |  |  |  |  |  |  |  |
| 4.Education | -.013 | -.049 | -.362** | ____ |  |  |  |  |  |  |  |  |
| 5.Number of children | .048 | .186** | .740** | -.304** | ____ |  |  |  |  |  |  |  |
| 6.Income | .007 | .008 | -.058 | .064 | -.047 | ____ |  |  |  |  |  |  |
| 7.Number of life time displacement | .019 | .024 | .196** | -.054 | .172** | -.060 | ____ |  |  |  |  |  |
| 8.Trauma score | .295** | .308** | .123* | -.028 | .102* | .113* | .025 | ____ |  |  |  |  |
| 9.Perceived social rejection | .403** | .581** | -.131 | .024 | .006 | .126 | .110 | .220 | ____ |  |  |  |
| 10.Enslavement events | .509** | .669** | -.111 | -.033 | .060 | .111 | .084 | .578** | .607** | ____ |  |  |
| 11.Number of family members directly affected by ISIS | .278** | .319** | .171** | -.023 | .114* | .010 | -.006 | .369** | .379** | .383** | ____ |  |
| 12.Enslavement duration | .515** | .531** | .054 | -.014 | .326* | -.142 | -.295 | .222 | .021 | .354* | .309 | ___ |
| Note: Spearman rank-order correlation coefficient was used.  * *p*< .05  ** *p*< .001 | | | | | | | | | | | | |
